# How Outcome Framing and Feedback Influence Decision Patterns over Time

**DOI:** 10.3390/bs16071079

**Published:** 2026-07-01

**Authors:** Maria Paula Armenta, Sébastien Hélie

**Affiliations:** Department of Psychological Sciences, Purdue University, West Lafayette, IN 47907, USA

**Keywords:** outcome framing, effortful decision-making, loss aversion, risk-taking

## Abstract

While loss aversion is a well-established phenomenon, less is known about how repeated feedback in gain or loss contexts changes effortful decisions over time. We hypothesized that repeated loss framing may reduce the willingness to take risks to avoid losses. To test this hypothesis, participants completed 108 trials in which they chose between performing an easy or a difficult task. The task was to stop a stopwatch as close as possible to three seconds. The hard task demanded greater precision and had a lower probability of success compared to the easy task. After each trial, participants received feedback on their performance and points, and at the end of the experiment, they rated their frustration level. Half the participants were randomly assigned to a gain condition while the remaining were assigned to a loss condition. In the gain condition, participants started with zero points and earned points after each trial. In the loss condition, participants began with a set number of points and lost points after each trial. Results showed that participants in the loss condition initially chose the hard task more frequently than those in the gain condition but gradually shifted toward the easy task over time. Participants in the loss condition also reported higher frustration levels at the end of the experiment. These findings suggest that framing and feedback-based learning may jointly shape how people balance effort, risk, and affect across repeated decisions.

## 1. Introduction

People often face decisions that require balancing potential rewards against risk. Choosing a challenging option can sometimes lead to greater gains but also carries a higher chance of failure. In this context, an important question is how people adjust their choices when outcomes are framed as gains or losses. Previous research has shown that people tend to prefer easier options, even when a more demanding choice could lead to better long-term outcomes (Kool et al., 2010; Shenhav et al., 2017). This reflects a general tendency to maximize rewards while minimizing costs, including both mental and physical effort (Lim et al., 2023). While mental effort can be difficult to quantify, tasks with lower performance accuracies (e.g., harder tasks) are often described as requiring more cognitive effort (e.g., Westbrook et al., 2013).

Research on effort-based decision-making shows that individuals adjust how much effort they invest depending on how valuable a reward seems and how likely they are to succeed (Shenhav et al., 2013). According to Frömer et al. (2021), when success feels achievable and the reward is meaningful, people are more willing to take on difficult tasks. In contrast, Crawford et al. (2023) explained that when tasks require more mental effort (e.g., harder tasks), people usually choose easier options unless they have a strong reason to select the harder task. This pattern aligns with the law of less work (Hull, 1943), which suggests that people prefer the less effortful path to reach a goal unless they have a strong reason to do otherwise. However, there are important individual differences in these trends (Randez & Hélie, 2024, 2026), and little work has been devoted to how this tendency is adjusted by feedback framing over time.

### 1.1. Framing

Effort and reward are not the only factors that shape decisions. The way a situation is framed—as a potential gain or loss—also influences decisions (Steiger & Kuhberger, 2018). According to Prospect Theory (Tversky & Kahneman, 1981), people evaluate outcomes relative to a reference point rather than by their absolute value. They tend to be more sensitive to losses than to equivalent gains, a phenomenon known as *loss aversion* (Sokol-Hessner & Rutledge, 2018). As a result, people often take greater risks to avoid losses than to pursue equal gains. While previous studies have focused on how framing affects risk-taking, less is known about how it shapes effortful choices and how these choices evolve as people learn from repeated feedback. The present study explored how outcome framing influences the decision to perform a more difficult task under uncertainty. Because the task involved continuous feedback, participants could gradually learn about the probabilities of success in each condition. Loss aversion has been shown to be extremely robust in previous research, and persists even in the presence of repeated feedback (Barron & Erev, 2003). However, its relationship with task difficulty and cognitive effort (both often measured by task performance) has been less explored. The present study aims to examine how framing and learning interact over time to influence effortful decision-making.

### 1.2. Learning About Risk

Learning from feedback plays a key role in how people adjust their behavior across repeated decisions. When outcomes are uncertain, individuals gradually update their expectations based on success and failure feedback (Behrens et al., 2007; Lim et al., 2025). This process can change not only how much risk or effort people are willing to take but also how confident they feel about their decisions. In tasks that involve both effort and uncertainty, feedback helps participants evaluate how much effort is “worth it” as they gain more experience. Therefore, studying framing in a repeated-feedback context allows for understanding not only initial responses to gains and losses but also how those responses evolve with learning.

Importantly, learning from repeated feedback is not purely cognitive. It is often accompanied by emotional reactions—especially when feedback involves failure or loss (Donaldson et al., 2016). One of the most common emotional responses in such contexts is frustration, which occurs when progress toward a goal is blocked (Roseman et al., 1994). Frustration may make decision-making feel more difficult and reduce motivation to keep trying (Harlé et al., 2023). As a result, affective responses may affect decision-making, and understanding this connection could help explain how framing effects develop over time. Although frustration may affect motivation (viz., willingness to work for a reward), in the present study, it was included as a secondary measure—an emotional factor that accompanies performance feedback but is not the main focus of our research.

In summary, this study examines how outcome framing (gain vs. loss) influences effortful decision-making under uncertainty and how feedback-based learning shapes these patterns over time. We also explore frustration as a complementary emotional response that may provide insight into participants’ experiences during the task.

### 1.3. Hypotheses

In the present experiment, participants were asked to repeatedly stop a stopwatch after exactly three seconds. In each trial, they could choose an easy (more error tolerance) or a hard (less error tolerance) trial. Half the participants would gain points on successful trials (with more points associated with hard trials) while the other half could only lose points (with a smaller loss associated with hard trials). We predicted that participants in the loss condition would choose hard trials more often than those in the gain condition. According to Prospect Theory (Tversky & Kahneman, 1981), people evaluate outcomes relative to a reference point, such as what they currently have or expect to have. In the loss condition, participants start with points and view each trial as a potential loss relative to that reference point, which may motivate them to take greater risks or invest more effort to avoid losing. In contrast, participants in the gain condition are expected to choose easier trials more often, since they start from zero and view each success as an opportunity to earn rather than to prevent a loss. We also predicted that participants in the loss condition would report higher levels of frustration than those in the gain condition. The repeated experience of losing points, even after performing well, may produce feelings of unfairness or reduced control, leading to higher emotional distress.

To anticipate our findings, participants in the loss condition initially chose difficult trials more often than those in the gain condition, indicating a higher willingness to take risks at the beginning of the experiment. However, as the task progressed, their preference for hard trials declined, suggesting that repeated exposure to losses reduced their motivation to exert effort. In contrast, participants in the gain condition showed a more stable pattern of choices across trials, with little change over time. Additionally, participants in the loss condition reported higher levels of frustration, reflecting the emotional impact of repeated losses.

## 2. Method

This experiment was pre-registered on the Open Science Framework (OSF) prior to data collection. The full pre-registration can be accessed at [https://osf.io/v2p3h (accessed on 27 June 2026)].

### 2.1. Participants

The study included 161 undergraduate students from a large midwestern university, all at least 18 years old, who were enrolled in an introductory psychology course and were recruited through SONA Purdue. This was a pre-registered convenience sample based on earlier work in our laboratory (Lim et al., 2023). After data exclusion described in Section 3, a post hoc power of *β* = 0.711 was obtained with an *α* = 0.05 to detect an interaction in the reported 2 × 4 mixed ANOVA on the number of hard choices. They were randomly assigned to one of four conditions: Group 1 Gain (*n* = 40), Group 2 Gain (*n* = 40), Group 3 Loss (*n* = 41), and Group 4 Loss (*n* = 40). In exchange for participation, students received partial course credit and had the opportunity to earn a performance-based bonus of up to $5, depending on their results in the experiment. 

### 2.2. Materials

**Figure 1:**
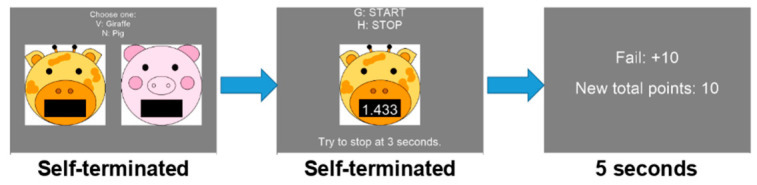
Step-by-step trial procedure. The stopwatches were represented by Giraffe and Pig images in the experiment. The watch counter appeared in the black rectangle. Feedback showed the points obtained in the current trial as well as cumulative points for the session.

The experiment was conducted using PsychoPy 2024.1.4. Participants took part in a decision-making task involving two stopwatches represented by a Giraffe and a Pig image (Figure 1). These images were used to distinguish between tasks of varying difficulty. Task difficulty was defined as the precision required to successfully stop the stopwatch (i.e., higher required precision meant a more difficult task).

### 2.3. Stopwatch Task

The stopwatch task was adapted from Murayama et al. (2015). The main differences between the current task and Murayama et al. were: (1) the time interval to stop the stopwatch was shorter (3 s vs. 5 s), (2) all trials included self-determined choices (i.e., there were no forced trials). Specifically, participants were asked to choose between two stopwatches, represented by a Giraffe and a Pig, by pressing specific keys on the keyboard. Pressing “V” displayed the Giraffe, while pressing “N” displayed the Pig. The assignment of difficulty was counterbalanced across participants, with the Giraffe being the difficult task for Group 1 Gain and Group 3 Loss, and the Pig being the difficult task for Group 2 Gain and Group 4 Loss.

Once participants had selected a stopwatch, they pressed the “G” key to start the stopwatch and the “H” key to stop it as close to 3 s as possible. The difficulty of the task depended on the precision required: for the easy task, participants succeeded if they stopped the stopwatch within a time window of 2.7 to 3.3 s, while for the difficult task, success required stopping the stopwatch within a narrower window of 2.98 to 3.02 s.

After each trial, participants received on-screen feedback indicating whether they succeeded or failed. This feedback included a visual message showing “Success” or “Failure” along with the number of points gained or lost based on their performance (see Table 1). The program continuously recorded participants’ task choices (easy or difficult), response times, points gained or lost during each trial, and their total points at the end of the experiment.

### 2.4. Frustration Rating Scale

At the end of the experiment, participants were asked to rate their frustration level on a scale from 0 (no frustration) to 10 (extremely frustrated).

### 2.5. Monetary Compensation

Participants were compensated based on the points they accumulated during the experiment, with a maximum possible payment of $5. We expected that the gain and loss conditions would end up with different point totals based on pilot experiments in our lab, so we created separate compensation scales for each condition depending on their total points earned. The pay scales are shown in Table 2.

### 2.6. Procedure

Participants began the study by signing a consent form and were seated at a computer to start the experiment. They were informed that they would be choosing between two stopwatches represented by a Giraffe and a Pig but were not told which stopwatch corresponded to the easy or difficult task. In the gain condition, participants had opportunities to win points, while in the loss condition, participants could only lose points. To avoid bias, participants were not informed of their condition assignment. The experiment consisted of 108 trials, with a break every 27 trials to reduce participant fatigue.

After stopping the stopwatch, participants received feedback immediately on the screen for 5 s. The feedback indicated whether they were successful (“Success” or “Failure”) and showed how many points they gained or lost. The feedback also displayed their total accumulated points. The total duration of each trial, including the time to make a choice and the feedback, was approximately 8–10 s, depending on the participant’s reaction time. The entire experiment lasted between 17 and 20 min, depending on how quickly each participant completed the tasks.

In the gain condition, participants could theoretically earn a maximum of 4320 points if they consistently succeeded on the difficult stopwatch task throughout all trials. In contrast, participants in the loss condition began with 4320 points and lost points based on their performance in each trial, as outlined in Table 1.

### 2.7. Design

This study used a 2 (Gain vs. Loss condition) × 2 (Easy vs. Difficult task) mixed factorial design. The two independent variables were condition assignment and task difficulty. Half of the participants had the Giraffe as the easy task and the Pig as the difficult task. In contrast, the other half had the Pig as the easy task and the Giraffe as the difficult task, ensuring counterbalancing.

## 3. Results

A total of 11 participants were excluded from the following analyses. Four participants were removed because they only selected one type of trial (either only easy or only hard). The remaining seven participants were excluded because they did not follow experimental instructions. This was determined as follows. For each participant, we calculated average accuracy separately for easy and hard trials. We then subtracted the mean hard trials accuracy from the mean easy trials accuracy. Participants who had negative values, which indicated better performance on hard trials than easy trials, were excluded. This was justified because the easy and hard trials only differed in the required timing precision of the button press. A precision sufficient to be successful in hard trials would always allow for success in the easy trials. The reverse, however, was not true. As a result, performing better in hard trials compared to easy trials suggests lapses in attention or that participants were not consistently trying their best. The final number of participants in each condition was: Group 1 Gain (*n* = 38), Group 2 Gain (*n* = 39), Group 3 Loss (*n* = 35), and Group 4 Loss (*n* = 38).

All statistics were calculated using R version 4.5.0. Factorial ANOVA were computed using the ‘rstatix’ package, while Bayes Factors (BF) were computed using the ‘BayesFactor’ package. In the following analyses, we report BF_10_ and use the strength of evidence table proposed by Kass and Raftery (1995) to interpret the results. Bayesian statistics were included to allow for interpreting null results.

### 3.1. Average Success Rates by Conditions

**Figure 2:**
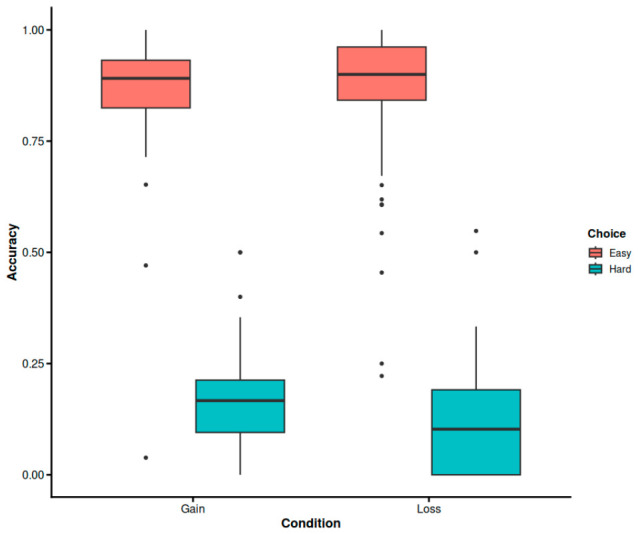
Average Success Rate in Easy and Hard Choices by Condition.

As shown in Figure 2, participants in both conditions had higher success rates for easy trials compared to hard trials. However, the condition did not seem to influence performance, as both gain and loss conditions showed similar success rates. To confirm these observations, we conducted a 2 (condition) × 2 (choice) mixed ANOVA on trial accuracy. Homogeneity of variance for the difference scores was assessed using Levene’s test and was not significant [*F*(1, 148) = 2.22, *p* = 0.138], indicating that the assumption was satisfied. The main effect of condition was not significant [*F*(1, 148) = 1.74, *p* = 0.189, *BF* = 0.367, *Not worth more than a bare mention*], indicating that the overall accuracy did not differ between the gain and loss conditions. However, there was a significant main effect of choice [*F*(1, 148) = 3364.85, *p* < 0.001, *BF* = 3.453 × 10^150^, *Decisive*], showing that participants were decisively more successful in easy trials (M = 87.57%) than in hard trials (M = 17.57%). The interaction effect between condition and choice was not significant [*F*(1, 148) = 1.92, *p* = 0.168, *BF* = 0.400, *Not worth more than a bare mention*], suggesting that the difference in success rates between easy and hard trials did not vary across the two framing conditions.

To further assess overall performance, we conducted an independent samples *t*-test comparing the final number of points earned between the gain and loss conditions. The assumption of homogeneity of variance was satisfied, as indicated by a non-significant Levene’s test [*F*(1, 148) = 1.66, *p* = 0.199]. The results showed a significant difference, [*t*(148) = 20.71, *p* < 0.001, *BF* = 7.963 × 10^44^, *Decisive*], indicating that participants in the gain condition (2809 points) finished the experiment with decisively more points than those in the loss condition (1757 points). This was expected based on pilot experiments conducted in our laboratory and justified the separate point conversion scales listed in Table 2. To ensure that the adjusted pay scale was successful in correcting this inequality, an independent samples *t*-test compared total money earned between the gain and loss conditions. The assumption of homogeneity of variance was again satisfied, as indicated by a non-significant Levene’s test [*F*(1, 148) = 1.31, *p* = 0.255]. Earnings did not differ between the gain and loss conditions, [*t*(148) = −0.710, *p* = 0.479, *BF* = 0.222, *Substantial*]. Overall, participants in both conditions earned an average of $3.45.

Lastly, an independent samples *t*-test was conducted to observe how frustration levels differed between conditions. The assumption of homogeneity of variance was satisfied, as indicated by a non-significant Levene’s test [*F*(1, 148) = 0.393, *p* = 0.532]. The *t*-test showed that participants in the loss condition reported significantly higher frustration levels than those in the gain condition, [*t*(148) = −3.0, *p* < 0.001, *BF* = 35.258, *Strong*]. The average level of frustration in the gain condition was 2.53, compared to the loss condition, which had 3.67. This finding supports the hypothesis that loss scenarios are more likely to be associated with greater frustration than gain scenarios, even when earning the same amount of money.

### 3.2. Average Number of Hard Choices and Trial Blocks

To understand how the selection of hard choices changed over time across the two conditions, a 4 (trial block) × 2 (condition) mixed ANOVA was conducted. Levene’s tests indicated that the homogeneity of variance assumption was met for Blocks 2–4, [all *Fs*(1, 148) ≤ 3.11, *ps* ≥ 0.080], but was violated for Block 1, [*F*(1, 148) = 5.77, *p* = 0.018]. As a result, the Greenhouse–Geisser correction was applied to the degrees of freedom where appropriate. The main effect of condition was not statistically significant [*F*(1, 148) = 1.183, *p* = 0.279, *BF* = 0.364, *Not worth more than a bare mention*], indicating that participants in the gain and loss conditions did not differ in their overall number of hard choices. However, the main effect of trial block was significant [*F*(2.36, 349.42) = 9.051, *p* < 0.001, *BF* = 1349.172, *Decisive*], showing that the number of hard choices changed over time, with participants choosing fewer hard trials as the blocks progressed.

**Figure 3:**
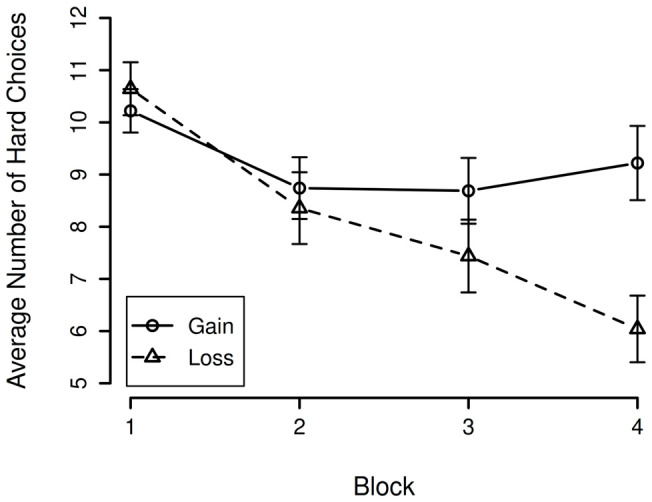
Average Number of Hard Choices in Trial Blocks by Condition.

However, both these main effects need to be interpreted in the context of a statistically significant interaction [*F*(2.36, 349.42) = 3.556, *p* = 0.023, *BF* = 1.466, *Not worth more than a bare mention*], indicating that changes in hard trials selection over time differed between the gain and loss conditions. As shown in Figure 3, participants in the loss condition had a significant decrease in hard choices across blocks [*F*(2.47, 178.1) = 12.347, *p* < 0.001, *BF* = 72,256.16. *Decisive*], whereas participants in the gain condition showed no significant change over time [*F*(2.25, 170.9) = 1.379, *p* = 0.254, *BF* = 0.087, *Strong*].

As shown in Figure 3, participants in the loss condition initially selected hard trials more often (M = 10.64) compared to those in the gain condition (M = 10.22). However, the loss condition’s selection of difficult trials declined steadily across blocks, reaching an average of 6.04 in the final block. In contrast, the gain condition showed a more stable pattern over time, with only small fluctuations and a slight increase in the last block (M = 9.22).

## 4. Discussion

This study examined how task framing (as a gain or a loss) affects risky decision-making. At the beginning of the experiment, participants in the loss condition chose hard trials more often, suggesting a willingness to accept greater risk to avoid losing points. However, as the experiment went on, they began choosing easier trials more frequently. This shift may reflect a combination of factors—repeated losses could have made participants more cautious about risky outcomes and less motivated to keep trying. In contrast, participants in the gain condition showed a more stable pattern over time, possibly because earning points felt more rewarding and less frustrating. Overall, these results suggest that loss framing can initially encourage risk-taking but may also reduce motivation when negative feedback continues.

The pattern observed in the gain condition is theoretically informative when interpreted in the context of the loss condition. Participants in the gain condition did not simply choose the easiest option throughout the task; rather, they showed a relatively stable willingness to select difficult trials across blocks. This stability contrasts with the loss condition, where participants initially selected difficult trials at a comparable or slightly higher rate but showed a marked decline over time. Thus, the gain frame appeared to support a more consistent balance between potential reward and task difficulty, whereas the loss frame may have initially promoted riskier, higher-effort choices but became less motivating as participants accumulated negative feedback. Importantly, this interpretation is consistent with the finding that participants in the loss condition reported greater frustration at the end of the experiment. Together, these results suggest that gain framing may help preserve engagement with challenging options, while loss framing may produce a more fragile form of motivation that is susceptible to erosion through repeated failure or negative feedback.

### 4.1. Implications

These findings support Prospect Theory, which proposes that people are more sensitive to losses than to gains. The results also extend this idea by showing that loss framing not only changes how people make risk-related choices but also how their behavior evolves over time. Similar to Harlé et al. (2023), our results suggest that repeated negative feedback can reduce motivation in decision-making, even when participants begin with a strong desire to avoid losses.

These findings also fit within a broader literature on effort-based decision-making, feedback learning, and affective responses to outcomes. Recent work suggests that decisions to exert effort depend not only on reward magnitude but also on perceived capability, task difficulty, and the probability of success (Randez & Hélie, 2024; Salamone & Correa, 2024). From this perspective, the decline in hard choices in the loss condition may reflect a learned reduction in the subjective value of the difficult option as participants experienced repeated failures or point losses. This interpretation is also consistent with recent evidence that risk-taking in experience-based decisions is shaped strongly by prior learning from gains and losses, rather than by static preferences alone (Erdman et al., 2025). Similarly, Buckley et al. (2025) found that loss framing in repeated real-effort tasks did not necessarily increase effort and was associated with higher stress, paralleling the present finding that loss framing produced greater frustration. More broadly, recent research indicates that gain- and loss-related choices may rely on partially dissociable processes (Bedder et al., 2023), suggesting that the gain and loss conditions in the present study should not be viewed as mirror images of one another. Instead, the present findings extend this literature by showing that outcome framing changes not only initial effortful risk-taking, but also the trajectory of effortful choice under repeated feedback.

Beyond these behavioral patterns, emotional factors may also help explain why performance declined under the loss frame. Another important finding was that participants in the loss condition reported higher levels of frustration than those in the gain condition. Although frustration was only measured at the end of the task, it served as a secondary measure to capture the emotional context of participants’ experiences, and this difference may suggest that repeated exposure to losses could have been emotionally taxing. These findings are consistent with the idea that negative feedback can influence motivation and effort over time. When people feel that their effort is not rewarded, motivation may decrease—a pattern that aligns with the decline in hard-task choices observed in the loss condition. However, because frustration was not measured continuously, this interpretation should be taken cautiously.

Taken together, these findings indicate that the way outcomes are framed can influence both the risks people take and how consistently they maintain challenging choices over time. Framing establishes the reference point from which outcomes are evaluated: in the gain condition, feedback signals progress toward reward, whereas in the loss condition, feedback signals the erosion of an initial endowment. Repeated feedback then allows participants to update the perceived value of the difficult option based on experienced success, failure, and emotional cost. Under loss framing, this process may create a feedback loop in which negative outcomes increase frustration, frustration reduces the subjective value of continued effort, and participants increasingly shift toward the easier option. Under gain framing, by contrast, feedback may be less affectively costly, allowing participants to maintain a more stable level of engagement with the difficult task. Thus, a central contribution of the present study is to show that framing effects in effortful decision-making are dynamic: they unfold through the interaction of reference-dependent valuation, learning from feedback, and affective responses to repeated outcomes.

### 4.2. Limitations and Future Research

One limitation of this study is that effort and reward magnitude were confounded in the task design. Hard trials required greater precision, which affected the probability of success and the potential amount of points gained or lost. Because of this, it is difficult to know whether participants’ choices were driven mainly by the effort required, by the expected value of the reward, or by both factors together. Future research could separate these variables to better understand how effort and reward independently influence decision-making.

A second limitation is that frustration was only measured at the end of the task, so we could not evaluate how it changed over time. One consequence of this limitation is that it is difficult to interpret whether the reduction in hard choices in the loss condition reflected an increase in frustration or a reduction in motivation because the participants were learning that they were unlikely to be successful. Future studies could include continuous emotional measures to better understand how motivation and emotion interact during repeated feedback.

A third limitation is that participants partook in a very simple laboratory task for points. While this simplification allowed for increased internal validity, this often comes at the cost of reduced ecological validity. Future research should replicate these findings in a more complex and realistic (“real-world”) setting where participants are directly receiving primary rewards (e.g., food) or money. Replicating similar patterns with real rewards instead of points in complex settings would greatly increase the potential applications of the present research.

Lastly, risk-taking has been shown to be affected by gender (Byrnes et al., 1999), so it is possible that men and women behaved differently in our study. We unfortunately did not collect demographic data, so we could not test for that effect. Participants were randomly recruited from a sex-balanced subject pool, so we expect that the experimental conditions were balanced, but we cannot know for sure. Future work should collect demographic information to ensure proper sex balance and possible differences in risk-taking behaviors.

## 5. Conclusions

Overall, this study highlights that loss framing can promote risk-taking early on but may lead to faster declines in motivation with continued negative feedback. These findings emphasize how feedback and framing together shape behavior across time.

## Figures and Tables

**Table 1:** Number of points earned or lost by trial.

	Gain Condition	Loss Condition
Succeed Easy Task	30	−20
Succeed Difficult Task	40	−10
Fail Easy Task	10	−40
Fail Hard Task	20	−30

**Table 2:** Money conversion rate in the gain and loss conditions.

Compensation	Points in the Gain Condition	Points in the Loss Condition
$1	0–2600	0–1500
$2	2601–2700	1501–1600
$3	2701–2800	1601–1700
$4	2801–2900	1701–1800
$5	2901<	1800<

## Data Availability

All the collected data can be accessed at https://osf.io/mgqkp/ (accessed on 27 June 2026).
